# Reference Genes for Expression Studies in Human CD8^+^ Naïve and Effector Memory T Cells under Resting and Activating Conditions

**DOI:** 10.1038/s41598-020-66367-1

**Published:** 2020-06-10

**Authors:** Marco Geigges, Patrick M. Gubser, Gunhild Unterstab, Yannic Lecoultre, Renato Paro, Christoph Hess

**Affiliations:** 1 Epigenomics Group, Department of Biosystems Science and Engineering, ETH Zürich, Basel, Switzerland; 2 Immunobiology Laboratory, Department of Biomedicine, University and University Hospital of Basel, Basel, Switzerland; 3 Faculty of Science, University of Basel, Basel, Switzerland; 4 Department of Medicine, University of Cambridge, Cambridge, UK

**Keywords:** Reverse transcription polymerase chain reaction, Gene expression, CD8-positive T cells

## Abstract

Reverse-transcription quantitative real-time polymerase chain reaction (RT-qPCR) is widely used for mRNA quantification. To accurately measure changing gene transcript levels under different experimental conditions, the use of appropriate reference gene transcripts is instrumental. In T cell immunology, suitable reference genes have been reported for bulk CD4^+^ and CD8^+^ T cells. However, many CD4^+^ and CD8^+^ T cell subsets have been described in the past. Although they respond differently to given activation stimuli, proper validation of suitable reference genes in these subsets is lacking. In this study, we evaluated twelve commonly used reference gene products in human naïve (NV) and effector memory (EM) CD8^+^ T cells under non-activated and activated (2 h, 10 h and 20 h) conditions. We used five different statistical approaches for data analysis. Our results show that a number of widely used reference transcripts become differentially expressed under activating conditions. Using them as references markedly alters results as exemplified with IFNG mRNA expression. The only candidate reference gene products that remained stable during the activation process were 18S rRNA and SDHA mRNA, encouraging their usage as reference gene products for RT-qPCR experiments, when quantifying mRNA levels in human NV and EM CD8^+^ T cells.

## Introduction

Due to its speed, sensitivity and simplicity, reverse-transcription quantitative real-time polymerase chain reaction (RT-qPCR) is widely used to quantify mRNA abundance under different experimental conditions in gene expression projects and validation studies of genome-wide high-throughput transcriptome analyses^1^. To produce reliable results, an appropriate normalization method is essential. Standardizing the biological starting material is sometimes difficult, if not impossible. However, even if an adjustment of the starting material like cellular count is reliable, alterations in RNA extraction, RNA integrity, reverse transcription and PCR amplification efficiency may still lead to erroneous quantification^2^. The use of internal reference genes for normalization can correct for these variations^3^. By definition, reference genes are expected not to change their expression in response to the experimental conditions under investigation. Genes that are universally required for basic cellular functions are thought to be stably expressed under various physiological and experimental conditions and thus often used to normalize target gene expression^4^. However, recent studies indicated that a number of commonly used reference genes do undergo significant regulation and become differentially expressed between tissues, cell types and cell subpopulations, or they may change expression during processes such as differentiation or activation^4,5^. Still, they are frequently used without proper validation of their expression stability in the investigated conditions. Many studies reported that the use of inappropriate reference genes may bias overall gene expression results and lead to incorrect conclusions^6–8^.

Studies that evaluate reference genes in human primary immune cells are rare. The expression stability of candidate genes has been investigated in bulk CD4^+^ and CD8^+^ T cells^9,10^, but to the best of our knowledge not in subpopulations of CD8^+^ T cells. However, naïve (NV) and effector memory (EM) CD8^+^ T cells are markedly different cell subtypes, distinctly responding to activation signals^11–13^.

Validated reference genes in both subtypes upon activation are lacking. In peripheral blood mononuclear cells, and bulk CD4^+^ and CD8^+^ T cells, 18S rRNA has been shown to be stably expressed following activation^9,14^. Its expression stability has, however, not been assessed in CD8^+^ T cell subsets.

This work characterizes the stability of twelve commonly used reference gene transcripts, including 18S rRNA, in CD8^+^ T cell subpopulations under resting and activating conditions. For validation, we investigated how interferon gamma (IFNG) mRNA expression was affected by normalization with these reference gene products.

## Results

### Evaluation of primer pairs, amplification specificity and efficiency

Primer pairs for twelve commonly used candidate reference gene products, and IFNG mRNA as a target gene induced by activation, were first evaluated (Table 1). Candidate reference genes were chosen based on the best reference genes reported earlier in human peripheral blood mononuclear cells (PBMCs)^14,15^, pan T cells (CD4^+^ and CD8^+^ combined)^16,17^, CD4^+^ T cells^9,10,18^ and CD8^+^ T cells^9,19^. Their specificity was confirmed by melting curve analysis and agarose gel electrophoresis of PCR products. Melting curves for all primer pairs were characterized by a single peak and no signal was present in negative controls (Supplementary Figure S1). Analysis also revealed single product-specific melting temperatures (Table 1). Single bands of appropriate size after agarose gel electrophoresis of PCR products additionally verified the specificity of the primers (Supplementary Figure S2). The primer efficiency and the associated correlation coefficient R^2^ were determined by the standard curve method using a pool of cDNA samples. Amplification efficiencies ran between 90.1% and 97.7% with correlation coefficients of at least 0.995 (Table 2). These results demonstrated that all primer pairs used in this study were suitable for RT-qPCR experiments.

**Table 1:** Overview of genes and primers used in this study.

Gene Symbol	Gene Name	Ensembl Gene ID	Primer Sequences	Size of PCR Product [bp]	Melting Point
ACTB	Actin beta	ENSG00000075624	F: 5′-CAA CCG CGA GAA GAT GAC CC-3′ R: 5′-AGA GGC GTA CAG GGA TAG CA-3′	94	82.1 °C
B2M	Beta-2-microglobulin	ENSG00000166710	F: 5′-TGT CTT TCA GCA AGG ACT GGT-3′ R: 5′-ATG CTG CTT ACA TGT CTC GAT-3'	147	81.4 °C
CASC3	Cancer susceptibility 3	ENSG00000108349	F: 5'-TGA AAG TGC AGA AGA CTC GGA-3 R: 5'-CTT TGC CTC TCT CCA GTC ACA-3'	168	80.6 °C
GAPDH	Glyceraldehyde-3-phosphate dehydrogenase	ENSG00000111640	F: 5'-GGT CAC CAG GGC TGC TTT TA-3' R: 5'-TTC CCG TTC TCA GCC TTG AC-3'	147	81.4 °C
HMBS	Hydroxy-methylbilane synthase	ENSG00000256269	F: 5'-TGA GAG TGA TTC GCG TGG GTA-3' R: 5'-GAA TCT TGT CCC CTG TGG TGG-3'	138	84.9 °C
HPRT1	Hypoxanthine phosphoribosyl-transferase 1	ENSG00000165704	F: 5'-GAC CAG TCA ACA GGG GAC AT-3' R: 5'-GCC TGA CCA AGG AAA GCA AAG-3'	133	78.9 °C
IPO8	Importin 8	ENSG00000133704	F: 5'-CCT CCA CCA GGA GAA GCA AT-3 R: 5'-TGG CAC GGA GAC ACA TTG TTA-3'	136	79.8 °C
PPIB	Peptidylprolyl isomerase B	ENSG00000166794	F: 5'-TGA AGA TGT AGG CCG GGT GA-3' R: 5'-CCG CCC TGG ATC ATG AAG TC-3'	153	81.1 °C
RPLP0	Ribosomal protein lateral stalk subunit P0	ENSG00000089157	F: 5'-TGT GGG AGC AGA CAA TGT GG-3' R: 5'-CCG GAT ATG AGG CAG CAG T-3'	163	86.4 °C
SDHA	Succinate dehydrogenase complex flavoprotein subunit A	ENSG00000073578	F: 5'-GCA TTT GGC CTT TCT GAG GC-3' R: 5'-CTC CAT GTT CCC CAG AGC AG-3'	117	82.1 °C
UBE2D2	Ubiquitin conjugating enzyme E2 D2	ENSG00000131508	F: 5'-TGA ATG ATC TGG CAC GGG AC-3' R: 5'-TCC ACC CTG ATA GGG ACT GT-3'	116	82.4 °C
18S rRNA	18S ribosomal RNA	NCBI ID: 106631781	F: 5'-CTC AAC ACG GGA AAC CTC AC-3' R: 5'-CGC TCC ACC AAC TAA GAA CG-3'	110	84.0 °C
IFNG	Interferon gamma	ENSG00000111537	F: 5'-GGC TTT TCA GCT CTG CAT CG-3' R: 5'-CGC TAC ATC TGA ATG ACC TGC-3'	115	79.2 °C

**Table 2:** Parameters derived from the RT-qPCR standard curve method for the primer pairs used in this study.

Gene Symbol	Intercept	Slope	PCR Amplification Efficiency	Regression Coefficient (R-squared)
ACTB	15.44	3.491	93.4	0.9996
B2M	14.09	3.482	93.7	0.9995
CASC3	21.88	3.519	92.4	0.9994
GAPDH	14.72	3.574	90.4	0.9993
HMBS	22.85	3.459	94.6	0.9990
HPRT1	23.18	3.535	91.8	0.9987
IPO8	22.38	3.579	90.3	1.0000
PPIB	19.16	3.510	92.7	0.9977
RPLP0	16.72	3.435	95.5	0.9983
SDHA	21.74	3.566	90.7	0.9996
UBE2D2	20.12	3.378	97.7	0.9996
18S rRNA	4.95	3.583	90.1	0.9957
IFNG	19.36	3.520	92.3	0.9992

### Evaluation of candidate reference genes’ expression in NV and EM CD8^+^ T cells upon 2 h activation

The expression of the twelve selected candidate reference gene products was measured by RT-qPCR in paired NV and EM human CD8^+^ T cell samples, both non-activated and activated for 2 h with anti-CD3/anti-CD28 monoclonal antibody-coated microbeads. The quantification cycles of the PCR reaction (Cq values) were distributed over a wide range from 6.2 to 29.6 across all analyzed samples, with mean Cq values between 6.9 ± 0.4 (Cq ± SD, 18S rRNA) and 28.5 ± 0.6 (HMBS). For all gene products, the Cq values per experimental condition followed a normal distribution.

**Figure 1:**
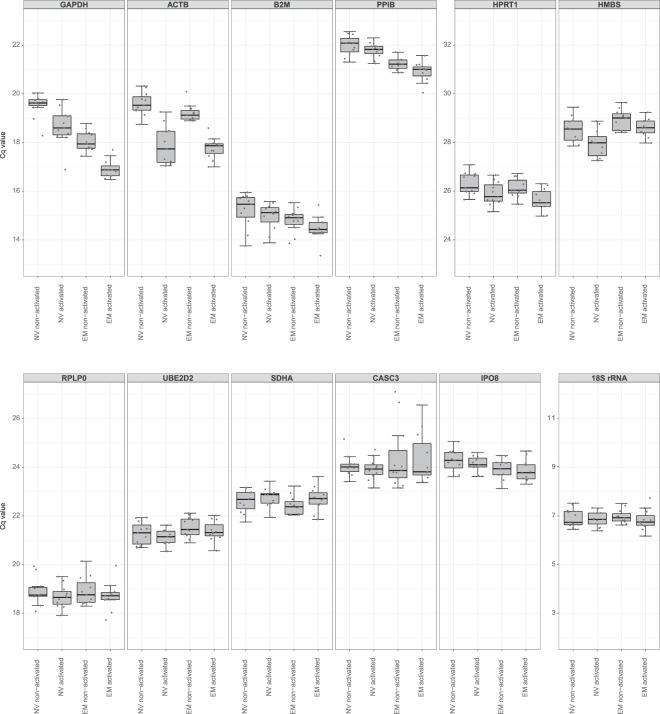
Cq values for twelve candidate reference gene products in NV and EM CD8^+^ T cells, both non-activated and activated for 2 h. Boxplots represent median and 25th and 75th percentiles (lower and upper hinges). Whiskers extend from the hinges to the smallest and largest value, respectively, no further than 1.5 times the inter-quartile range from the hinges. Sample number: n = 11.

The Cq values of all candidate gene products were tested for differences between the experimental subgroups NV and EM cells, non-activated or activated for 2 h each. For each gene product, the four groups were pairwise compared using linear mixed effect models. Cq values for ACTB and GAPDH mRNAs changed significantly (adjusted p-value < 0.05) by more than 0.5 Cq units upon activation in both cell types (Fig. 1 and Supplementary Table S1). On top, Cq values for GAPDH mRNA differed also between the two T cell subsets. HPRT1 mRNA expression levels were similarly affected by activation, especially in EM cells. PPIB mRNA expression differed between the two cell types in non-activated and activated states, while different Cq values were found for HMBS mRNA expression upon activation in NV cells and between activated NV and EM cells. These differences in Cq values suggested potential differential regulation of the affected genes upon activation and/or in the two cell types, which were therefore excluded from further analysis. For B2M, CASC3, IPO8, RPLP0, SDHA, UBE2D2 and 18S rRNA, no strong evidence of differential expression between the experimental subgroups was found, thus meeting the conditions required for suitable reference genes. These remaining seven candidate gene products covered Cq values between 14.9 ± 0.6 (B2M) and 24.2 ± 0.9 (CASC3), except for 18S rRNA with a mean Cq value of 6.9 ± 0.4.

### Analysis of expression stability of reference gene products in the 2 h activation dataset

To further analyze the expression stability of the selected reference gene products, five different statistical approaches were used: NormFinder^20^, BestKeeper^21^, ΔCq^22^, geNorm^23^ and its derivative method ΔCq*M^24^. Gene products with significantly different Cq values (adjusted p-value < 0.05, absolute difference > 0.5 Cq units) between experimental subgroups (ACTB, GAPDH, HMBS, HPRT1 and PPIB) were excluded from further investigation. This was necessary, as particularly the analysis of expression stability by NormFinder requires that a gene’s average expression level is approximately the same in different experimental groups^20^. Additionally, the Cq values for 18S rRNA were much lower than the ones for the other remaining candidate reference mRNAs (Fig. 1). Therefore, heterogeneous variance is expected between the candidate genes with very different expression levels and Cq values. As a Pearson correlation coefficient must not be used in such cases^21^, BestKeeper analysis with all genes was restricted to the evaluation of Cq variation and 18S rRNA was excluded for subsequent correlation coefficient analysis.

Results for the tested candidate reference gene products from the five statistical methods are summarized in Table 3. All seven gene products showed low NormFinder values indicative of constant expression. NormFinder ranked 18S rRNA and IPO8 mRNA as the most stable gene products, followed by RPLP0, UBE2D2, SDHA, B2M and CASC3 for the 2 h activation time point. IPO8 and UBE2D2 were identified by NormFinder as the best combination of two genes for normalization with a combined stability value of 0.08 (Supplementary Table S2).

**Table 3:** Analysis of expression stability of reference gene products with different statistical approaches.

Rank	NormFinder		ΔCq method		BestKeeper		geNorm		ΔCq*M	
	Gene	Stability Value	Gene	Mean SD	Gene	SD	Gene	Stability Value M	Gene	Comprehensive ΔCq*M Ranking
1	18S rRNA	0.12	IPO8	0.54	18S rRNA	0.29	IPO8	0.367	18S rRNA	1.86
2	IPO8	0.18	18S rRNA	0.57	UBE2D2	0.33	SDHA	0.373	RPLP0	2.45
3	RPLP0	0.19	UBE2D2	0.57	IPO8	0.35	18S rRNA	0.376	IPO8	2.66
4	UBE2D2	0.20	SDHA	0.58	SDHA	0.36	UBE2D2	0.403	SDHA	3.41
5	SDHA	0.22	RPLP0	0.66	RPLP0	0.38	B2M	0.459	UBE2D2	4.00
6	B2M	0.29	B2M	0.72	B2M	0.49	RPLP0	0.503	CASC3	4.70
7	CASC3	0.33	CASC3	0.99	CASC3	0.65	CASC3	0.626	B2M	6.48

The results from the ΔCq approach were similar (Supplementary Table S3, Supplementary Figure S3). ΔCq variability was minimal for IPO8 mRNA. 18S rRNA was ranked as second most stable reference gene product and UBE2D2, SDHA and RPLP0 mRNAs followed with only marginal higher mean standard deviations in ΔCq values.

BestKeeper analysis based on standard deviation revealed 18S rRNA and UBE2D2 as the transcripts with the least variability. As all tested gene products showed a considerably low SD value, all of them were included into the subsequent repeated pairwise correlation analysis, except for 18S rRNA due to the heterogenous variance issue. The correlation coefficient to the BestKeeper index calculated without 18S rRNA was highest for IPO8 and UBE2D2 mRNAs, followed by SDHA and RPLP0 mRNAs (Supplementary Table S4).

geNorm analysis rated IPO8 and SDHA mRNAs as the most stable gene products and 18S rRNA, UBE2D2, B2M, RPLP0 and CASC3 mRNAs were assigned to the succeeding positions. The geNorm algorithm was also used to determine the optimal number of reference gene products required for the normalization of gene expression. The pairwise variation between the sets of the most stable two and three gene products, respectively, V2/3, was 0.112 (Supplementary Figure S4). As this value is lower than the threshold of 0.15, the optimal number of reference gene products to be combined into a normalization factor would be two according to geNorm.

Using the geNorm stability value and taking group differences into account, comprehensive ranking according to the ΔCq*M values identified 18S rRNA as the overall best suitable reference gene product. For some specific pairwise comparisons of experimental groups, other gene products provided a smaller group bias (Supplementary Table S5, Supplementary Table S6). In the comprehensive ΔCq*M ranking, considering all biologically relevant pairwise comparisons of experimental groups, RPLP0 mRNA followed as the second most appropriate reference gene product. IPO8, SDHA, UBE2D2, CASC3 and B2M mRNAs were ranked subsequently.

To combine the results from these five statistical approaches, the geometric mean of the ranks, which were assigned to the gene products by the individual methods, was determined and used for an overall comprehensive ranking (Supplementary Table S7). Accordingly, 18S rRNA was ranked as the overall best reference gene product for expression studies in NV and EM human CD8^+^ T cells, both non-activated and activated for 2 h. 18S rRNA was the top reference gene product according to NormFinder, BestKeeper and the ΔCq*M approach, ranked second by the ΔCq method and on third position according to geNorm. Comprehensive ranking listed IPO8 as the second most appropriate reference gene product. IPO8 was among the top two reference gene products in the analysis with three out of five applied methods and on third position according to the other two approaches. UBE2D2, SDHA, RPLP0, B2M and CASC3 mRNAs followed in the comprehensive ranking.

Although a ranking is provided, all seven candidate gene products that were analyzed are appropriate reference gene products in this experimental setting with an activation period of 2 h. BestKeeper considers genes with SD > 1 to be instable^21^. In this dataset, even CASC3 mRNA, the gene product with the highest SD among the seven tested genes, had an SD according to BestKeeper clearly below this proposed stability threshold (Table 3). The suitability of all seven genes as reference genes was confirmed by the geNorm analysis. M values below 0.5 are indicative of high expression stability, while M values up to 1 are accepted in more heterogeneous samples^25^. In this dataset, the M values were below 0.5 for most analyzed genes, thus demonstrating their high stability (Table 3). RPLP0 with 0.503 was slightly above this threshold and CASC3 (0.626), the gene product with the highest M value, was also clearly below 1.

### Evaluation of candidate reference genes’ expression upon 10 h activation

**Figure 2:**
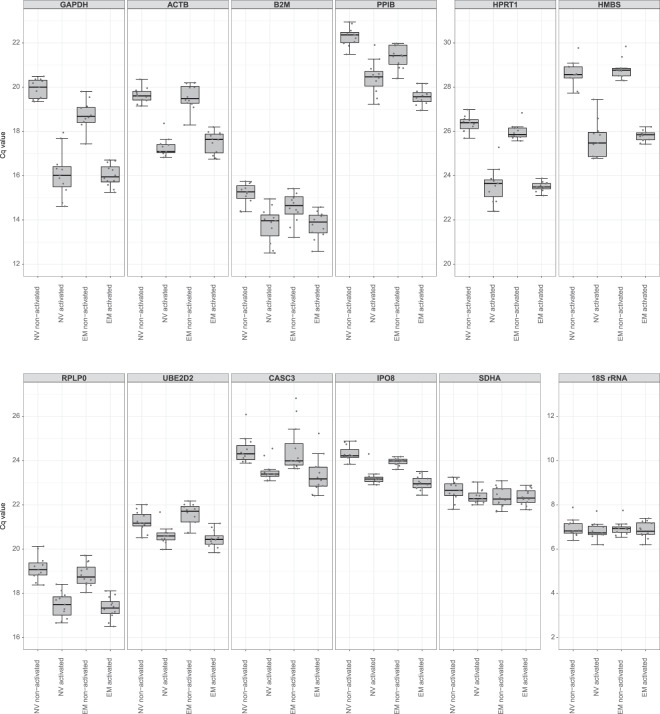
Cq values for twelve candidate reference gene products in NV and EM CD8^+^ T cells, both non-activated and activated for 10 h (further details as in legend of Fig. 1). Sample number: *n* = 11–12.

Similarly to the 2 h time point, the expression of the twelve candidate reference genes was also measured by RT-qPCR in NV and EM CD8^+^ T cells activated for 10 h. Differences between experimental subgroups were again analyzed with linear mixed effects models. All genes with significantly different Cq values between experimental subgroups in the 2 h activation dataset were neither stable in the 10 h activation data. ACTB, GAPDH, HMBS, HPRT1 and PPIB mRNAs showed significantly lower Cq values, for example, in both cell types upon activation for 10 h (Fig. 2, Supplementary Table S8). On top, five out of the seven reference gene products, that were stable after 2 h of activation, were substantially changed after this longer activation period. B2M, CASC3, IPO8, RPLP0 and UBE2D2 mRNAs had all significantly lower Cq values after 10 h of activation, regardless of the cell type, thus making them unsuitable as reference gene products at later activation time points. Only 18S rRNA and SDHA mRNA were constantly expressed, even after an activation period of 10 h. Having just two stable candidate gene products left, the algorithms used to further rank and evaluated the non-differentially expressed gene products in the 2 h activation dataset could not be applied to the 10 h activation data. For a useful analysis, it would be required to include more than these two stable genes into the calculations. However, the application of NormFinder, the ΔCq approach, geNorm and the ΔCq*M method requires as basic prerequisites that candidate genes are not differently expressed between subgroups or not co-regulated with other candidates, for example^20,22^. Consequently, these analyses cannot be extended to the genes with significantly different Cq values between experimental groups. Only BestKeeper’s SD analysis for a candidate gene is independent of the other genes and their potential expression change or co-regulation. Therefore, BestKeeper analysis was performed on the entire 10 h dataset and confirmed that 18S rRNA and SDHA mRNA are the least variable gene products and hence suited as proper reference genes in long-term activated CD8^+^ T cell subsets (Supplementary Table S9).

### Evaluation of candidate reference genes’ expression upon 20 h activation

**Figure 3:**
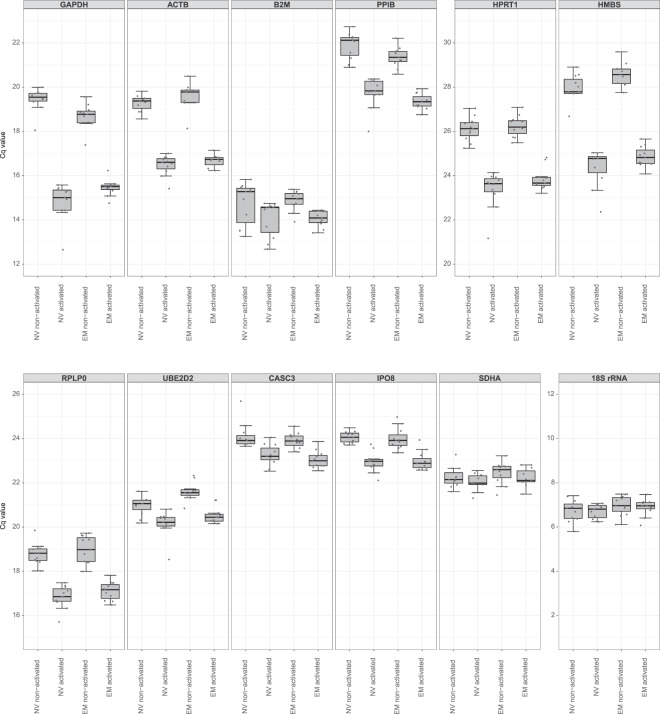
Cq values for twelve candidate reference gene products in NV and EM CD8^+^ T cells, both non-activated and activated for 20 h (further details as in legend of Fig. 1). Sample number: *n* = 11.

To assess the expression stability after even longer activation periods, the twelve candidate gene products were additionally analyzed in NV and EM CD8^+^ T cells that had been activated for 20 h. The same effects as upon 10 h of activation were found in the 20 h activation RT-qPCR dataset. ACTB, GAPDH, HMBS, HPRT1 and PPIB mRNAs, which were affected by 2 h and 10 h activation, had also significantly lower Cq values in both cell types upon activation for 20 h (Fig. 3, Supplementary Table S10). Similarly, B2M, CASC3, IPO8, RPLP0 and UBE2D2 mRNAs, which were stably expressed in the 2 h activation dataset, but changed significantly upon 10 h of activation, were also altered by 20 h of activation. In contrast, no difference was observed for 18S rRNA and SDHA mRNA upon activation for 20 h. This finding was supported by BestKeeper analysis (Supplementary Table S11), further recommending them as reference genes for long activation periods.

### Validation of reference gene products by analysis of target gene expression upon 20 h activation

To further assess the reliability of the selected reference gene products for normalization of RT-qPCR data in this experimental setting, the expression level of IFNG mRNA, which is highly induced upon activation in T cells, was additionally included in the RT-qPCR analysis. To illustrate the effects of normalization with an inappropriate reference gene product, IFNG mRNA expression was normalized to the two reference gene products found to be suitable reference gene products in the 20 h activation dataset, 18S rRNA and SDHA mRNA, and to the two mRNAs that varied most upon 20 h of activation, GAPDH and HMBS (Supplementary Table S10).

**Figure 4:**
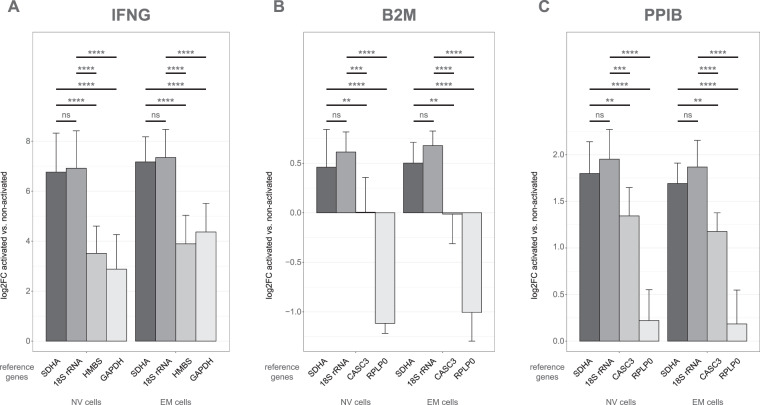
Effect of normalization with different reference gene products on (**A**) IFNG, (**B**) B2M and (**C**) PPIB mRNA expression upon 20 h of activation. IFNG, B2M and PPIB mRNA levels, respectively, were normalized to the indicated reference gene products. For both cell types, log2 fold changes for activated cells were calculated relative to the expression level in the corresponding non-activated cells. Mean values from eight donors are given. Error bars represent standard deviations. Non-significant (ns) *p* >  0.05, *p ≤ 0.05, **p < 10^–3^, ***p < 10^–5^, ****p < 10^–8^. Sample number: n = 8.

When normalized to SDHA mRNA or 18S rRNA, the log2 fold change (log2FC) of INFG mRNA induction in NV cells upon 20 h activation was 6.8 and 6.9, respectively (Fig. 4A). This large IFNG mRNA increase was underestimated when the expression was normalized to HMBS or GAPDH mRNAs. For the IFNG mRNA upregulation in NV cells, a log2FC of only 2.9 was reported when normalized to GAPDH mRNA, for example. This corresponded to a 16-fold smaller IFNG mRNA induction when GAPDH was used a reference gene product as compared to 18S rRNA. A similar effect of underrating IFNG mRNA induction was seen in EM cells, when expression was normalized to GAPDH or HMBS mRNAs instead of SDHA mRNA or 18S rRNA. Thus, accurate evaluation of the expression of inflammatory cytokines and other target genes in both NV and EM cells activated for 20 h depends on the usage of stable reference gene products, such as SDHA mRNA or 18S rRNA, for normalization.

Further analysis has been done by looking at the expression of B2M and PPIB mRNAs. Upon 20 h of activation, B2M mRNA expression was mildly increased in both NV and EM cells, when SDHA mRNA or 18S rRNA were correctly used for normalization (Fig. 4B). However, this induction was not observed when CASC3 or RPLP0 mRNAs, which were among the suitable reference gene products after 2 h of activation, were used to normalize expression in the 20 h activation dataset. When using CASC3 mRNA as reference, no change of B2M mRNA expression was reported upon activation in both cell types. Additionally, despite B2M mRNA being induced, its expression was incorrectly shown to be even markedly decreased when it was normalized to RPLP0 mRNA. PPIB mRNA expression was upregulated nearly 4-fold upon activation in both cell types, when correctly normalized to SDHA mRNA or 18S rRNA levels (Fig. 4C). In contrast, barely any induction was observed, when RPLP0 mRNA, for example, was inappropriately used for normalization, thus highlighting again the significance of evaluating and using proper reference gene products in any experimental setup.

## Discussion

To identify appropriate internal reference gene products for normalization of RT-qPCR data in T cell subsets is a challenge since (i) quiescent T cell subsets are distinct and (ii) further and uniquely change fundamental cellular processes upon activation^26–29^.

In this study, we evaluated the expression stability of twelve commonly used reference gene products in CD8^+^ T cell subpopulations under resting and activating conditions. We included not only NV and EM cells separately, but also looked at different activation periods of 2 h, 10 h and 20 h. We thoroughly assessed potential reference gene expression with five different statistical methods. To combine the results of these different algorithms, we defined an overall ranking based on the geometric mean of the stability ranks from the individual methods. Of course, this approach does not account for the strengths and limitations of each method. However, it is difficult to weight their results differently according to their power in each experimental setup and hence the geometric mean of individual ranks is commonly used to obtain a comprehensive ranking. Indeed, this reflected the results from the different algorithms in our 2 h activation dataset quite well. We found that 18S rRNA, IPO8, UBE2D2, SDHA, RPLP0, B2M and CASC3 mRNAs are appropriate gene products for normalization after 2 h activation. In contrast, only SDHA mRNA and 18S rRNA remained reliable reference gene products for 10 h and 20 h activation periods, respectively.

18S rRNA has been reported earlier as an appropriate reference RNA under activated conditions in PBMCs^14^, bulk CD4^+^ and CD8^+^ T cells^9^ and pan T cells (CD4^+^ and CD8^+^ combined)^16^. We extend this knowledge and found that 18S rRNA is also one of the appropriate reference gene products that is stably expressed in NV and EM CD8^+^ T cell subpopulations at all activation time points analyzed. However, it should be mentioned that there are some complications associated with its use. Ribosomal transcripts are usually much more abundant than target gene mRNAs, the rRNAs are transcribed by another polymerase than mRNAs and absent in selectively purified mRNA samples. Still, they are routinely used for normalization in many species-specific analyses and experimental settings^4^.

As an alternative in T cell subpopulations, we identified SDHA mRNA as a stable reference gene product throughout the activation process. SDHA mRNA has been described earlier as a possible reference gene product in human activated pan T cells^16^, but was described to be unsuitable for normalization in stimulated CD4^+^ T cells^18^. We broadened the knowledge by confirming stable expression also in CD8^+^ T cell subsets, namely NV and EM CD8^+^ T cells after activation. To our best knowledge, these studies are the only ones assessing SDHA mRNA as a possible reference gene product under activating conditions in T cells.

It is noteworthy that, in contrast to our own results, Ledderose and colleagues have not found a significant upregulation of IPO8 and GAPDH mRNAs upon 24 h activation. However, they used pan T cells for their analysis. In addition to our own results, other studies have confirmed the induction of GAPDH mRNA expression after activation in bulk CD8^+^ T cells^9^.

Induction of gene expression upon activation for ACTB, HMBS, HRPT1 and PPIB mRNAs is in line with results published earlier for related cell types and these transcripts are therefore not suitable as reference gene products for studies comparing activated with non-activated T cells^14,16^.

The use of inappropriate reference gene products for normalization causes wrong interpretations of data^6–8^. If our RT-qPCR dataset is normalized to RPLP0 mRNA, a transcript that is upregulated after activation, B2M mRNA seems to be downregulated. However, with the use of the appropriate reference gene products SDHA mRNA or 18S rRNA, a slight induction of B2M mRNA is measured (Fig. 4B). This underpins the importance of studies that evaluate candidate reference gene products in the specific experimental setting used.

Using five different statistical approaches, our study comprehensively expands the current basis about appropriate reference gene products in human T cell samples. We confirm a stable expression of SDHA mRNA not only in pan T cell samples, but also in NV and EM CD8^+^ T cell subsets. Our work forms a solid reference for future studies comparing gene expression in NV and EM CD8^+^ T cells. If RT-qPCR is used to quantify mRNA levels in human NV and EM CD8^+^ T cells, we recommend SDHA mRNA and 18S rRNA as transcripts for normalization.

## Methods

### Blood donors and ethical consent

All experiments and all methods in this study were performed in accordance with relevant guidelines and regulations. Human blood was obtained with ethical approval of the Red Cross, which was confirmed by the Ethical Committee of both Cantons of Basel, Switzerland. All experimental protocols performed in this study are standard and not subject to approval. Blood samples were obtained from healthy male and female donors (18–65 years old) as buffy coats after written informed consent (Blood Donor Center, University Hospital Basel, Switzerland).

### Peripheral blood CD8^+^ T cell isolation and sorting

PBMCs were isolated from healthy blood donors by standard density-gradient centrifugation protocols (Lymphoprep Fresenius Kabi, Norway). CD8^+^ T cells were enriched by positive selection using magnetic CD8^+^ beads (Miltenyi Biotec, Germany). Cells were rested overnight prior to cell sorting in R10 RPMI-1640 supplemented with 10% fetal bovine serum (FBS). For sorting of NV and EM CD8^+^ T cells, cells were stained with anti-CD62L (ImmunoTools, Germany) and anti-CD45RA (Beckman Coulter, USA) antibodies. NV and EM CD8^+^ T cells were identified as CD62L^+^ CD45RA^+^ and CD62L^–^ CD45RA^–^ populations, respectively. Cell sorting was performed with a BD influx cell sorter (BD Bioscience, USA). Cells were rested in R10 for 2–4 h at 37 °C prior to activation.

### T cell activation

Human CD8^+^ T cell activation was performed with in-house generated anti-CD3/anti-CD28 monoclonal antibody-coated microbeads. Polybead microspheres (4.5 μm, Polyscience, Germany) were incubated with 99 μg anti-human CD28 IgG1 antibody (CD28.2 Biolegend, USA) and 1.0 μg anti-human CD3 IgG2a antibody (HIT3a, Biolegend, USA) or left unloaded for unactivated samples. 2*10^6^ sorted cells were activated in 1 ml R10FBS in a 24 well plate with a 2:1 ratio of beads to cells. Cells were harvested after 2 h, 10 h or 20 h activation by washing once with ice-cold PBS and subsequent resuspension in 1 ml ice-cold TRIzol reagent (Thermo Fisher Scientific, USA). Samples were stored at −80 °C until RNA isolation. No sample was stored longer than 2 months.

### RNA extraction and cDNA synthesis

RNA was isolated using the RNeasy Mini Kit (Qiagen, Germany). All samples were treated with DNase (Turbo DNA-free Kit, Thermo Fisher Scientific, USA) as recommended by the manufacturer. Integrity of isolated RNA was assessed on a 2100 Bioanalyzer System (Agilent Technologies, USA) and RNA concentration was measured with the Quant-iT RiboGreen RNA Assay Kit (Thermo Fisher Scientific, USA). cDNA was synthesized from 342 ng total RNA, reflecting the maximum possible amount from the least concentrated sample, in a total reaction volume of  20 µL, using the GoScript Reverse Transcriptase Mix (Promega, USA) with random primers according to the manufacturer’s instructions. Negative controls without the reverse transcriptase (minus RT controls) were processed in parallel. cDNA samples were stored maximally 2 days at −20 °C prior to quantitative RT-qPCR.

### Quantitative RT-PCR

Quantitative real-time PCR was performed with the GoTaq qPCR Master Mix (Promega, USA) and gene-specific, intron-spanning primer pairs (Table 1) on an Applied Biosystems ViiA 7 instrument (Thermo Fisher Scientific, USA). All primers were designed with NCBI Primer BLAST and synthesized by Microsynth, Switzerland. All reactions were run in duplicates in 384 well plates (Thermo Fisher Scientific, USA), including melting curve analysis. For exact pipetting, an Assist Plus Pipetting Robot (Integra Biosciences, Switzerland) was used. Negative controls without any template were processed in parallel and did not result in any qPCR signal. Specificity of primer pairs was verified by electrophoresis on a 2% (w/v) agarose gel. For visualization, the SYBR Safe DNA Gel Stain (Thermo Fisher Scientific, USA) was used in combination with the Gel Doc XR+ System (Biorad, USA). The expected product size was confirmed by using a 100 bp DNA Ladder (New England BioLabs, USA). For all primer pairs, standard curves were generated based on a five-fold dilution series of a pool of different cDNAs from samples analyzed in this work. Amplification efficiencies and correlation coefficients for each primer pair were calculated from the slopes of the standard curves using R version 3.5.1^30^.

### Statistical analysis

The Kolmogorov-Smirnov test was used to determine whether the distribution of Cq values per gene and experimental condition was normal. To identify differentially expressed genes between experimental subgroups, a linear mixed effects model with two crossed fixed effects (cell type, activation) with two levels each including their interaction and donor-dependent activation was used. For each activation dataset (2 h, 10 h, 20 h), calculations were carried out separately in R version 3.6.2^30^ and package lme4^31^. Pairwise comparisons between subgroups were performed for each gene with package emmeans^32^ and Tukey adjusted p-values were reported. Genes were considered significantly differentially regulated in two groups, if there was an adjusted p-value < 0.05 and an absolute value of the effect size > 0.5 Cq units, which is the range of Cq value variation that was observed within an experimental subgroup.

### Determination of reference gene expression stability

The stability of candidate gene products was evaluated with five different methods: NormFinder^20^, BestKeeper^21^, ΔCq^22^, geNorm^23^ and ΔCq*M method^24^.

NormFinder calculates an expression stability value for each gene product, considering not only intra- but also inter-group variation between sample subgroups. High expression stability of a transcript is characterized by a low stability value. NormFinder was used as an R script (NormFinder for R, version 5, 2015-01-05). As input, linear relative values were calculated from the raw Cq values following the comparative Cq method deltaCq^33^. Thereby, the amplification efficiency was taken into account (Table 2) and the sample with the lowest Cq value was used as a reference Cq value^34^.

The ΔCq method directly compares relative expression of pairs of genes within each sample to find stable reference genes. For this approach, the differences between the Cq values of all pairs of genes within each sample were calculated in R and evaluated by its standard deviation following the original description of this method^22^.

BestKeeper is an Excel-based spreadsheet software tool that uses standard deviation (SD) and repeated pairwise correlation analysis to evaluate the candidate reference gene products. The SD values are used to rank the gene products. Additionally, from the gene products with low standard deviations and high Pearson correlation coefficients, the BestKeeper index is computed as the geometric mean of the genes’ Cq values. Subsequently, the correlation between each candidate gene transcript and the BestKeeper index is calculated. A high coefficient of correlation with the BestKeeper index also indicates stable gene expression. BestKeeper was used as an Excel macroinstruction (version 1) and raw Cq values as well as amplification efficiencies were used as input values.

The geNorm algorithm uses raw Cq values and amplification efficiencies and calculates an expression stability measure M as the average pairwise variation of a certain transcript with all other candidate gene products. Following stepwise exclusion of the gene product with the highest M value, new M values are recalculated for the remaining gene products and used to rank them. The gene with the lowest M value is the one most stably expressed. The algorithm also allows for the determination of the optimal number of reference gene mRNAs to be used for normalization of gene expression. To this end, the pairwise variation V between two sets of genes, that contain an increasing number of genes, is determined. The set of genes with a V value below 0.15, which consists of the smallest number of genes, is considered an appropriate set of reference genes.

The ΔCq*M method uses the geNorm calculations and additionally assesses differences between experimental subgroups. The difference of Cq values between two groups is multiplied by the geNorm M value for each gene product. This ΔCq*M value is calculated for each pairwise comparison between subgroups and used to rank the genes. A comprehensive ranking by the ΔCq*M method is achieved by ordering the genes according to the geometric mean of the individual ranking positions of all pairwise comparisons.

To combine the results from all five methods, the geometric mean of the ranks, which were assigned to the genes by the individual approaches, was calculated and used to obtain an overall comprehensive ranking.

### Validation of reference gene products

To confirm the reliability of the selected reference gene transcripts, IFNG, B2M and PPIB mRNA expression was normalized to the two most stable and to two unstable reference gene products upon 20 h of activation in both NV and EM cells. Using the 2^-ΔΔCq^ method with the determined amplification efficiencies, relative fold changes were calculated between the activated and non-activated state in both cell types for each donor^33,35^ and Tukey adjusted p-values from linear mixed effects model analyses were reported.

## Supplementary information

Supplementary Information.

## Data Availability

Raw data is available upon request.
