# Supplementary material for: Reference Genes for Expression Studies in Human CD8+ Naïve and Effector Memory T Cells under Resting and Activating Conditions
Source: Sci Rep. 2020 Jun 10;10:9411. doi: 10.1038/s41598-020-66367-1 (PMC7286888; doi:10.1038/s41598-020-66367-1)
Supplement: Supplementary file 1 — Supplementary Information. [file 41598_2020_66367_MOESM1_ESM.pdf]

Supplementary Information

Reference Genes for Expression Studies in Human CD8$^+$ Naïve and Effector Memory T Cells under Resting and Activating Conditions

Marco Geigges [1, 2 #], Patrick M. Gubser [2 #], Gunhild Unterstab [2], Yannic Lecoultre [2], Renato Paro [1, 3 *], Christoph Hess [2, 4 *]

# Marco Geigges and Patrick M. Gubser contributed equally to this work.

* Renato Paro und Christoph Hess jointly supervised the work. Correspondence to Renato Paro (renato.paro@bsse.ethz.ch) and Christoph Hess (christoph.hess@usb.ch).

1 Epigenomics Group, Department of Biosystems Science and Engineering, ETH Zürich, Basel, Switzerland
2 Immunobiology Laboratory, Department of Biomedicine, University and University Hospital of Basel, Basel, Switzerland
3 Faculty of Science, University of Basel, Basel, Switzerland
4 Department of Medicine, University of Cambridge, Cambridge, UK

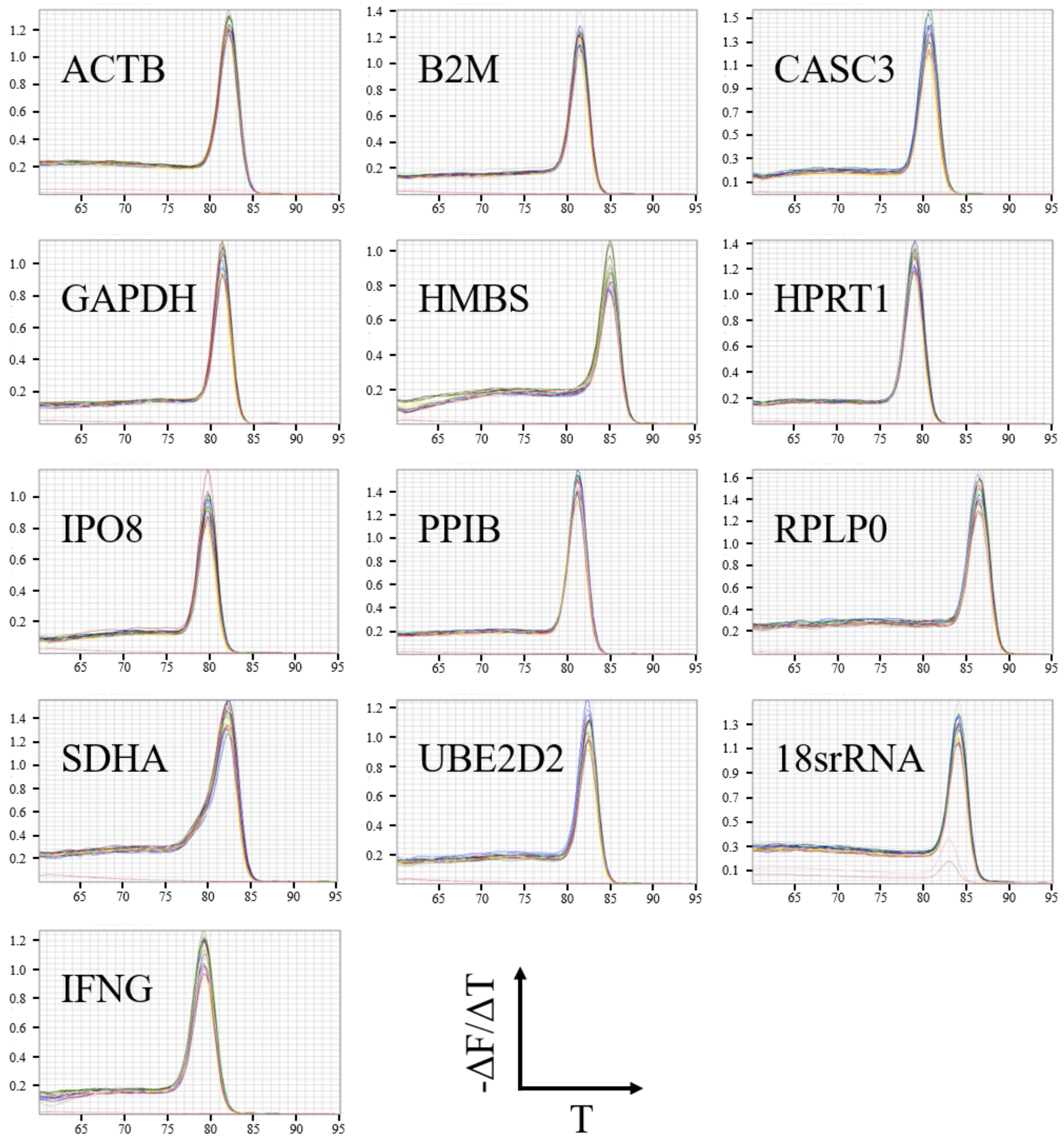

Supplementary Figure S1

Melting curve analysis for all primer pairs used in this study.

The melting curves of the amplification products have been visualized using the Applied Biosystems ViiA 7 instrument (-ΔF/ΔT (fluorescence change/temperature change) vs. temperature (T)). Specificity of the used primer pairs have been confirmed by a single peak in the melting curve. No peak was detected in the negative control samples.

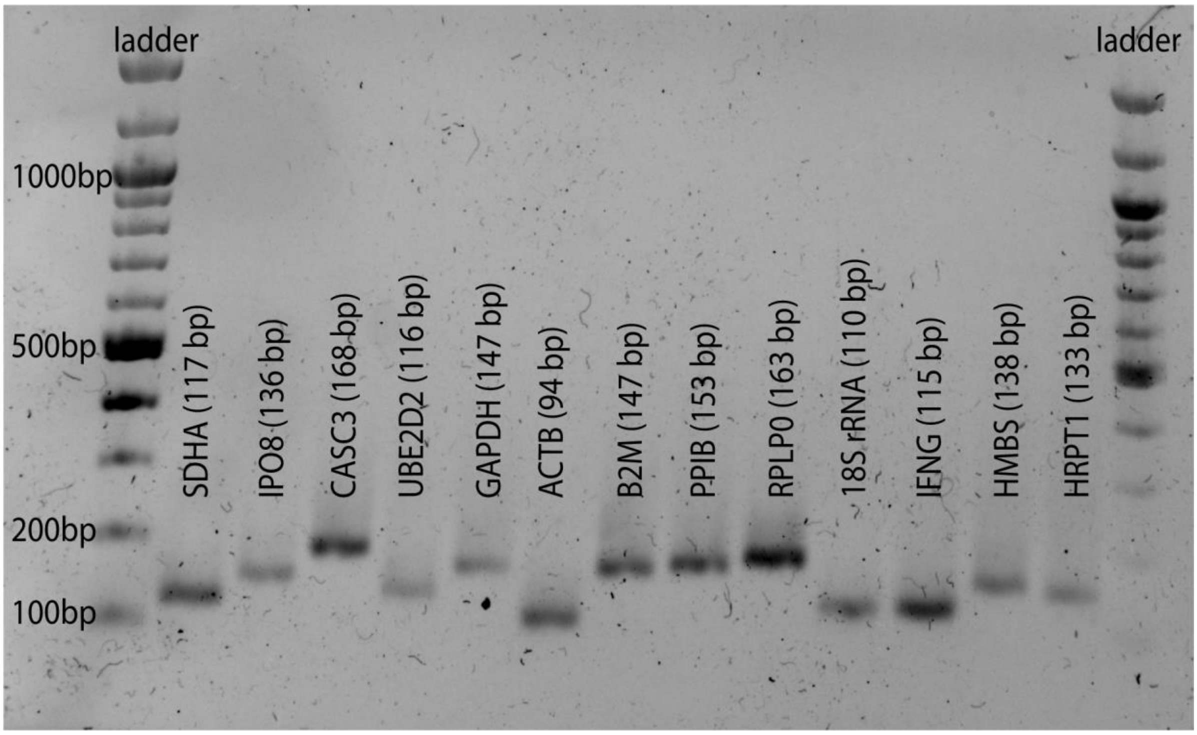

Supplementary Figure S2

Verification of primer specificity by agarose gel electrophoresis of PCR products.

Specificity of primer pairs was verified by electrophoresis on a 2% (w/v) agarose gel. Pooled DNA samples have been run and single bands have been visualized using SYBR Safe DNA Gel Stain. The expected product sizes (given in brackets) have been confirmed using a 100 bp DNA ladder.

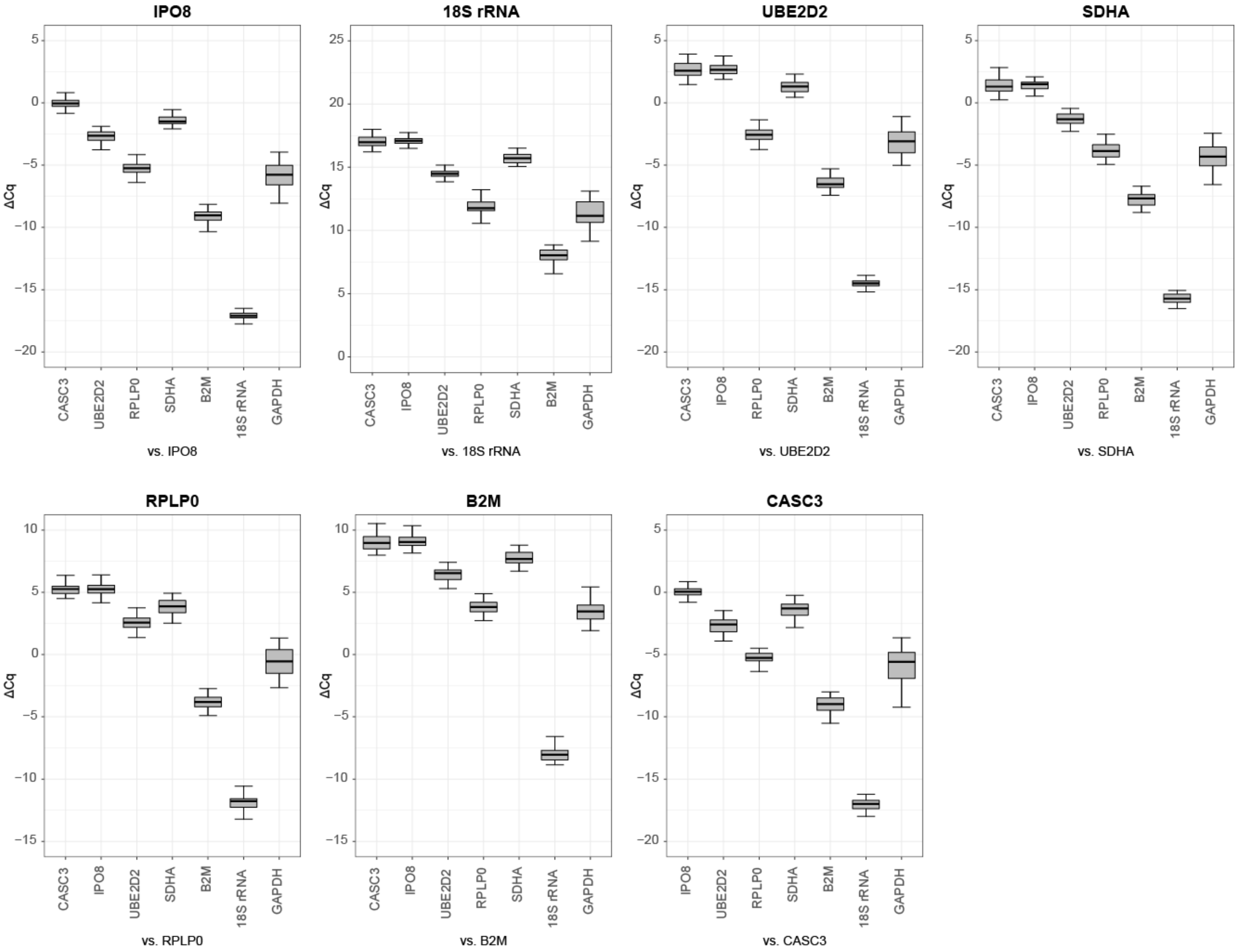

Supplementary Figure S3

ΔCq method results of the seven candidate gene products that were not differentially expressed between experimental subgroups in the 2 h activation dataset.

ΔCq variability in candidate reference gene products is shown as differences between the Cq values of the indicated pairs of gene products within each sample. For comparison, ΔCq for GAPDH mRNA, one of the transcripts that was differentially regulated between subgroups, is included into the graph.

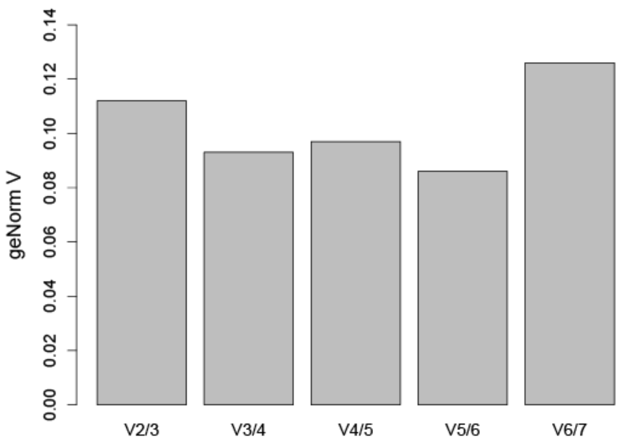

Supplementary Figure S4

Determination of the optimal number of reference gene products by the geNorm algorithm in the 2 h activation dataset.

Pairwise variation $V_{n/n+1}$ between two sets of genes with the n and n+1 most stable candidate reference gene products was calculated by geNorm. For V values below 0.15, the inclusion of the additional n+1th reference gene transcript is not required.

Supplementary Table S1

Pairwise comparisons of the Cq values of all candidate reference gene mRNAs between experimental subgroups of the 2 h activation dataset using linear mixed effects models

contrast	**NV_no_activ - EM_no_activ**		**NV_no_activ - NV_activ**		**EM_no_activ - EM_activ**		**NV_activ - EM_activ**	
gene	estimate	p value	estimate	p value	estimate	p value	estimate	p value
**ACTB**	0.317	0.155	1.754	0	1.424	4.51E-14	-0.013	1.000
**B2M**	0.299	0.069	0.243	0.125	0.319	0.020	0.375	0.008
**CASC3**	0.012	1.000	0.383	0.097	0.041	0.994	-0.329	0.106
**GAPDH**	1.565	0	0.864	4.97E-09	1.178	4.19E-14	1.879	0
**HMBS**	-0.451	0.013	0.570	2.16E-04	0.302	0.127	-0.719	1.07E-05
**HPRT1**	0.140	0.499	0.407	0.001	0.516	9.72E-06	0.249	0.069
**IPO8**	0.323	0.043	0.141	0.598	0.068	0.932	0.250	0.149
**PPIB**	0.717	1.21E-09	0.197	0.232	0.315	0.014	0.834	9.12E-13
**RPLP0**	0.131	0.887	0.258	0.422	0.194	0.660	0.067	0.979
**SDHA**	0.142	0.713	-0.149	0.624	-0.269	0.132	0.023	0.998
**UBE2D2**	-0.301	0.080	0.133	0.667	0.165	0.493	-0.269	0.109
**18S rRNA**	-0.113	0.770	0.029	0.995	0.139	0.647	-0.002	1.000

Supplementary Table S2

Combined stability values for all combinations of two genes for the 2 h activation dataset identified by NormFinder

**gene 1**	**gene 2**	**combined stability**
SDHA	IPO8	0.15
SDHA	CASC3	0.21
SDHA	UBE2D2	0.13
SDHA	B2M	0.19
SDHA	RPLP0	0.16
SDHA	18S rRNA	0.14
IPO8	CASC3	0.17
**IPO8**	**UBE2D2**	**0.08**
IPO8	B2M	0.22
IPO8	RPLP0	0.14
IPO8	18S rRNA	0.12
CASC3	UBE2D2	0.24
CASC3	B2M	0.20
CASC3	RPLP0	0.21
CASC3	18S rRNA	0.19
UBE2D2	B2M	0.16
UBE2D2	RPLP0	0.17
UBE2D2	18S rRNA	0.14
B2M	RPLP0	0.17
B2M	18S rRNA	0.16
RPLP0	18S rRNA	0.12

Supplementary Table S3

ΔCq method results for the 2 h activation dataset including mean differences between the Cq values of pairs of gene transcripts and standard deviations

vs.	IPO8		18S rRNA		UBE2D2		SDHA		RPLP0		B2M		CASC3		mean SD
	mean ΔCt	SD	mean ΔCt	SD	mean ΔCt	SD	mean ΔCt	SD	mean ΔCt	SD	mean ΔCt	SD	mean ΔCt	SD	
**IPO8**			-17.12	0.40	-2.68	0.46	-1.41	0.39	-5.26	0.55	-9.14	0.60	0.16	0.85	**0.54**
**18S rRNA**	17.12	0.40			14.44	0.42	15.71	0.42	11.87	0.63	7.98	0.61	17.28	0.92	**0.57**
**UBE2D2**	2.68	0.46	-14.44	0.42			1.27	0.47	-2.57	0.53	-6.46	0.52	2.84	1.01	**0.57**
**SDHA**	1.41	0.39	-15.71	0.42	-1.27	0.47			-3.85	0.65	-7.73	0.55	1.57	1.00	**0.58**
**RPLP0**	5.26	0.55	-11.87	0.63	2.57	0.53	3.85	0.65			-3.88	0.74	5.42	0.87	**0.66**
**B2M**	9.14	0.60	-7.98	0.61	6.46	0.52	7.73	0.55	3.88	0.74			9.30	1.27	**0.72**
**CASC3**	-0.16	0.85	-17.28	0.92	-2.84	1.01	-1.57	1.00	-5.42	0.87	-9.30	1.27			**0.99**

Supplementary Table S4

BestKeeper results for the 2 h activation dataset including standard deviation (SD) and coefficient of variance (CV)

Gene	SD	CV [% Cp]	coefficient of correlation
18S rRNA	0.29	4.13	n. d.
UBE2D2	0.33	1.54	0.680
IPO8	0.35	1.46	0.787
SDHA	0.36	1.61	0.647
RPLP0	0.38	2.00	0.646
B2M	0.49	3.30	0.592
CASC3	0.65	2.68	0.402

Supplementary Table S5

ΔCq*M values for biologically relevant comparisons between experimental subgroups in the 2 h activation dataset

comparison groups	ΔCq*M						
	18S rRNA	RPLP0	IPO8	SDHA	UBE2D2	CASC3	B2M
NV_no_activ - NV_activ	0.013	0.111	0.051	0.056	0.059	0.091	0.118
EM_no_activ - EM_activ	0.051	0.104	0.021	0.100	0.065	0.023	0.144
NV_no_activ - EM_no_activ	0.022	0.003	0.131	0.062	0.102	0.202	0.192
NV_activ - EM_activ	0.016	0.010	0.101	0.017	0.095	0.270	0.218

Supplementary Table S6

Ranking of candidate reference gene mRNAs according to their ΔCq*M values (Supplementary Table S5) for biologically relevant comparisons between experimental subgroups in the 2 h activation dataset

comparison groups	ranking according to ΔCq*M						
	1	2	3	4	5	6	7
NV_no_activ - NV_activ	18S rRNA	IPO8	SDHA	UBE2D2	CASC3	RPLP0	B2M
EM_no_activ - EM_activ	IPO8	CASC3	18S rRNA	UBE2D2	SDHA	RPLP0	B2M
NV_no_activ - EM_no_activ	RPLP0	18S rRNA	SDHA	UBE2D2	IPO8	B2M	CASC3
NV_activ - EM_activ	RPLP0	18S rRNA	SDHA	UBE2D2	IPO8	B2M	CASC3
comprehensive ranking based on geometric mean of individual ranking positions	18S rRNA<br>1.86	RPLP0<br>2.45	IPO8<br>2.66	SDHA<br>3.41	UBE2D2<br>4.00	CASC3<br>4.70	B2M<br>6.48

Supplementary Table S7

Ranking of candidate reference gene products in the 2 h activation dataset according to all different methods used

Gene	Ranking according to					
	NormFinder	ΔCq method	BestKeeper	geNorm	ΔCq*M	overall comprehensive ranking
18S rRNA	1	2	1	3	1	1.43
IPO8	2	1	3	1	3	1.78
UBE2D2	4	3	2	4	5	3.44
SDHA	5	4	4	2	4	3.64
RPLP0	3	5	5	6	2	3.90
B2M	6	6	6	5	7	5.97
CASC3	7	7	7	7	6	6.79

Supplementary Table S8

Pairwise comparisons of the Cq values of all candidate reference gene mRNAs between experimental subgroups of the 10 h activation dataset using linear mixed effects models

contrast	NV_no_activ - EM_no_activ		NV_no_activ - NV_activ		EM_no_activ - EM_activ		NV_activ - EM_activ	
gene	estimate	p value	estimate	p value	estimate	p value	estimate	p value
**ACTB**	-0.032	0.994	2.353	0	2.067	0	-0.318	0.050
**B2M**	0.615	0.007	1.428	5.63E-12	0.809	1.47E-04	-0.003	1.000
**CASC3**	-0.096	0.965	0.950	2.10E-05	1.161	2.22E-08	0.115	0.938
**GAPDH**	1.235	1.31E-10	3.867	0	2.685	0	0.053	0.993
**HMBS**	-0.159	0.812	2.989	0	3.011	0	-0.137	0.875
**HPRT1**	0.379	0.072	2.798	0	2.499	0	0.080	0.962
**IPO8**	0.331	0.026	1.062	2.95E-14	0.967	4.07E-14	0.236	0.200
**PPIB**	0.864	1.65E-09	1.861	0	1.836	0	0.839	1.33E-08
**RPLP0**	0.286	0.305	1.680	4.17E-14	1.501	3.35E-14	0.106	0.923
**SDHA**	0.260	0.096	0.216	0.258	-0.052	0.968	-0.007	1.000
**UBE2D2**	-0.341	0.027	0.622	9.99E-07	1.093	3.02E-14	0.131	0.696
**18S rRNA**	0.004	1.000	0.105	0.907	0.079	0.952	-0.023	0.999

Supplementary Table S9

BestKeeper results for the 10 h activation dataset including standard deviation (SD) and coefficient of variance (CV)

Gene	SD	CV [% Cp]
18S rRNA	0.27	3.97
SDHA	0.34	1.52
UBE2D2	0.53	2.54
IPO8	0.54	2.27
CASC3	0.68	2.84
B2M	0.69	4.82
RPLP0	0.81	4.46
PPIB	1.00	4.79
ACTB	1.11	6.00
HPRT1	1.33	5.34
HMBS	1.50	5.53
GAPDH	1.65	9.31

Supplementary Table S10

Pairwise comparisons of the Cq values of all candidate reference gene mRNAs between experimental subgroups of the 20 h activation dataset using linear mixed effects models

contrast	**NV_no_activ - EM_no_activ**		**NV_no_activ - NV_activ**		**EM_no_activ - EM_activ**		**NV_activ - EM_activ**	
gene	estimate	p value	estimate	p value	estimate	p value	estimate	p value
**ACTB**	-0.232	0.348	2.797	0	2.879	0	-0.150	0.670
**B2M**	0.249	0.127	0.698	2.76E-10	0.758	5.46E-12	0.308	0.024
**CASC3**	0.170	0.729	0.861	1.93E-04	0.887	1.09E-04	0.197	0.603
**GAPDH**	0.861	1.13E-09	4.586	0	3.273	0	-0.453	0.006
**HMBS**	-0.416	0.011	3.616	0	3.700	0	-0.332	0.076
**HPRT1**	0.106	0.811	2.719	0	2.469	0	-0.144	0.652
**IPO8**	0.088	0.892	1.125	3.69E-14	1.019	3.69E-14	-0.018	0.999
**PPIB**	0.717	4.56E-07	2.108	0	2.015	0	0.623	2.17E-05
**RPLP0**	0.152	0.341	1.978	0	1.873	0	0.048	0.951
**SDHA**	-0.162	0.639	0.228	0.295	0.221	0.289	-0.168	0.604
**UBE2D2**	-0.517	1.16E-04	0.809	2.04E-09	1.117	3.35E-14	-0.209	0.346
**18S rRNA**	-0.139	0.408	0.045	0.976	0.056	0.954	-0.128	0.441

Supplementary Table S11

BestKeeper results for the 20 h activation dataset including standard deviation (SD) and coefficient of variance (CV)

Gene	SD	CV [% Cp]
18S rRNA	0.34	4.95
SDHA	0.37	1.65
CASC3	0.47	2.01
UBE2D2	0.54	2.61
IPO8	0.56	2.39
B2M	0.57	3.95
RPLP0	0.93	5.19
PPIB	1.01	4.88
HPRT1	1.24	4.95
ACTB	1.41	7.84
HMBS	1.79	6.74
GAPDH	1.89	11.03
